# Diverse Basis of β-Catenin Activation in Human Hepatocellular Carcinoma: Implications in Biology and Prognosis

**DOI:** 10.1371/journal.pone.0152695

**Published:** 2016-04-21

**Authors:** Hirohisa Okabe, Hiroki Kinoshita, Katsunori Imai, Shigeki Nakagawa, Takaaki Higashi, Kota Arima, Hideaki Uchiyama, Toru Ikegami, Norifumi Harimoto, Shinji Itoh, Takatoshi Ishiko, Tomoharu Yoshizumi, Toru Beppu, Satdarshan P. S. Monga, Hideo Baba, Yoshihiko Maehara

**Affiliations:** 1 Department of Gastroenterological Surgery, Graduate School of Life Sciences, Kumamoto University, Kumamoto, Japan; 2 Department of Multidisciplinary Treatment for Gastroenterological Cancer, Kumamoto University Hospital, Kumamoto, Japan; 3 Department of Pathology, University of Pittsburgh, Pittsburgh, Pennsylvania, United States of America; 4 Department of Medicine, University of Pittsburgh, Pittsburgh, Pennsylvania, United States of America; 5 Department of Surgery and Science, Graduate School of Medical Sciences, Kyushu University, Fukuoka, Japan; University of Medicine, Greifswald, GERMANY

## Abstract

**Aim:**

β-catenin signaling is a major oncogenic pathway in hepatocellular carcinoma (HCC). Since β-catenin phosphorylation by glycogen synthase kinase 3β (GSK3β) and casein kinase 1ε (CK1ε) results in its degradation, mutations affecting these phosphorylation sites cause β-catenin stabilization. However, the relevance of missense mutations in non-phosphorylation sites in exon 3 remains unclear. The current study explores significance of such mutations in addition to addressing the clinical and biological implications of β-catenin activation in human HCC.

**Methods:**

Gene alteration in exon3 of *CTNNB1*, gene expression of β-catenin targets such as *glutamate synthetase (GS)*, *axin2*, *lect2* and *regucalcin (RGN)*, and protein expression of β-catenin were examined in 125 human HCC tissues.

**Results:**

Sixteen patients (12.8%) showed conventional missense mutations affecting codons 33, 37, 41, and 45. Fifteen additional patients (12.0%) had other missense mutations in codon 32, 34, and 35. Induction of exon3 mutation caused described β-catenin target gene upregulation in HCC cell line. Interestingly, conventional and non-phosphorylation site mutations were equally associated with upregulation of β-catenin target genes. Nuclear localization of β-catenin was associated with poor overall survival (*p* = 0.0461). Of these patients with nuclear β-catenin localization, loss of described β-catenin target gene upregulation showed significant poorer overall survival than others (*p* = 0.0001).

**Conclusion:**

This study suggests that both conventional and other missense mutations in exon 3 of *CTNNB1* lead to β-catenin activation in human HCC. Additionally, the mechanism of nuclear β-catenin localization without upregulation of described β-catenin target genes might be of clinical importance depending on distinct mechanism.

## Introduction

Primary liver cancer which is predominantly hepatocellular carcinoma (HCC), is the sixth most common cancer worldwide and the third most frequent cause of cancer-related mortality [1]. β-Catenin gene (*CTNNB1*) mutation and mutation in *AXIN1* are major oncogenic gene alterations in HCC, seen in 20–40% and 10%, respectively [2]. *CTNNB1* mutations cause stabilization of β-catenin protein through loss of codon 33, 37, 41, and 45 phosphorylation sites for glycogen synthase kinase 3β (GSK3β) and casein kinase 1ε (CK1ε) resulting in upregulation of β-catenin target genes such as glutamine synthetase (GS), axin2 and regucalcin [3–6].

In addition to mutations directly affecting phosphorylation sites, there are a substantial number of patients who have other missense mutation sites in exon 3 (codon 32, 34, 35, and 36) [7]. These mutations in *CTNNB1* exon 3 are theoretically considered to affect β-catenin signaling because of the structural change of protein caused by alteration of amino acid close to the GSK3β binding. Austinat et al. reported that transfection of P44A or H36P, mutants of *CTNNB1* that are not direct phosphorylation sites of CK1ε or GSK3β enhanced the promotor activity of TCF4/β-catenin [8]. However, this has not been investigated directly in HCC patients.

In the current study we validate that missense mutations not directly affecting GSK3β or CKε binding sites in β-catenin gene indeed show active β-catenin signaling in human HCC. We also show that those HCC that exhibit nuclear β-catenin localization have worse prognosis. Immunohistochemical and transcriptomic analysis revealed that some patients whose tumor showed nuclear β-catenin localization but didn’t have determined target gene upregulation turned out to show the poorest survival. We eventually discuss the implication of targeting β-catenin signaling in patients with aberrant β-catenin activation in HCC caused by genetic alterations [9, 10].

## Materials and Methods

### Clinical Tissue Samples

One hundred twenty five patients with HCC were registered and underwent curative first-line surgery at the Department of Gastroenterological Surgery, Kumamoto University Hospital, between 2005 and 2010. Specimens of primary HCC and adjacent normal liver tissues were obtained from the patients after their written informed consent was obtained. This study was approved by the Human Ethics Review Committee of the Graduate School of Life Sciences, Kumamoto University (Kumamoto, Japan).

### RNA Extraction and Quantitative Reverse Transcriptase-Polymerase Chain Reaction (qRT-PCR)

RNA extraction, reverse transcription, and qRT-PCR was performed as previously described [11]. Primers were designed using the Roche webpage and the Universal Probe Library in accordance with the manufacturer’s recommendations. The primers used were as follows: *Axin2* forward 5′- GCTGACGGATGATTCCATGT -3′, *Axin2* reverse 5′- ACTGCCCACACGATAAGGAG -3′, and universal probe #56; *Lect2* forward 5′- GCTATCCATGGAATATTAGAACTTGA -3′, *Lect2* reverse 5′- TGCATCTCTCATTTCTTTAGTGTGA -3′, and universal probe #58; *Regucalcin* forward 5′- TCAATGATGGGAAGGTGGAT -3′, *Regucalcin* reverse 5′- TGGTGCCGCTCAAGAACT -3′, and universal probe #30; *GS* forward 5′- AAGAGCGGAGCGTGTGAG -3′, *GS* reverse 5′- TCATGGTGGAAGGTGTTCTG -3′, and universal probe #55; HPRT forward 5′-TGACCTTGATTTATTTTGCATACC-3′, HPRT reverse 5′- CGAGCAAGACGTTCAGTCCT-3′, and universal probe #73. HPRT was used as internal control gene. For amplification, an initial denaturation at 95°C for 10 min was followed by 15 s at 95°C, 15 s at 60°C, and 13 s at 72°C.

### DNA extraction and Direct sequencing

Genomic DNA was extracted using QIAamp DNA Micro Kit (Qiagen). Amplification exon 3 of *CTNNB1*, extraction of PCR products, and sequencing are performed as previously described [12].

### Immunocytochemistry

Cultured cells were washed, and fixed in 4% paraformaldehyde for 15 min. Subsequently, the samples were washed and nonspecific protein binding sites were blocked by 30 min incubation in 3% bovine serum albumin. Sections were incubated for 1 h with rabbit anti-β-catenin antibody (SantaCruz). After 3 washes in PBS, the sections were incubated for 30 min at room temperature in the dark, with the appropriate secondary antibody. After 2 washes in PBS, Hoechst staining was performed and the mounting reagent was applied.

### Luciferase Assay

Hep3B-S33Y and Hep3B-pCI cells were plated in 24 well plate. TCF4 reporter plasmid was transfected with lipofectamin 2000 (Invitrogen) into them. Cells were lysed and prepared by using the luciferase reporter assay system (Promega, Madison, WI). Activity was read on a luminometer (Lumat; EG & G Berthold).

### Statistical Analysis

Statistical analyses were performed using JMP ver. 8.0 (SAS Institute., Cary, NC, USA). Values are expressed as the mean ± SD. Differences between groups were calculated by the Wilcoxon test. P < 0.05 was defined as significant.

## Results

### Gene alterations in exon 3 of CTNNB1

Direct sequencing analysis of exon3 of *CTNNB1* revealed 16 patients with conventional missense mutations in the GSK3β and CK1ε phosphorylation sites affecting codons 33, 37, 41, and 45. Additional 15 patients showed mutations affecting neighboring codons that were not direct phosphorylation sites. Thus overall, 31 (26.9%) patients had *CTNNB1* mutations exon 3, which included transitions, transversions, and two very short deletions affecting codon 33 (S1 Fig and S1 Table).

### β-Catenin target gene expressions are regulated by CTNNB1 mutation in human HCC cell line

**Fig 1:**
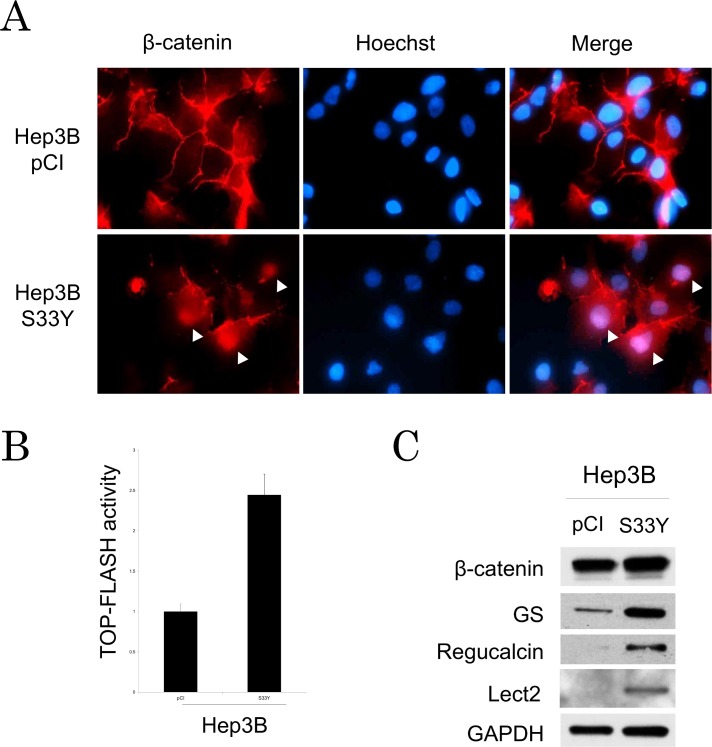
Regulation of β-catenin target genes by *CTNNB1* exon3 mutation in human HCC cell line. A. Stable cell line expressing *CTNNB1* exon3 mutation (Hep3B-S33Y) is established. Control cell line is shown as Hep3B-pCI. Immunofluorescent staining is performed with β-catenin antibody. Nuclear staining is performed with Hoechst33342. Arrow heads show nuclear translocation of β-Catenin. B. TOP-Flash activity is compared between Hep3B-S33Y and Hep3B-pCI. C. β-catenin target protein expressions (GS, Regucalcin and Lect2) of Hep3B-S33Y and Hep3B-pCI are shown by Western blot.

Since we previously demonstrated gene candidates which β-catenin directly regulates in mouse liver [13]. Among those candidates, we picked up 4 genes such as *Axin2*, *Regucalcin*, *Lect2* and *GS*. To confirm that those genes are regulated by *CTNNB1* mutation in human HCC, we established stable cell line expressing mutant β-catenin (S33Y) using Hep3B cell line. Hep3B transfected with S33Y (Hep3B-S33Y) shows nuclear translocation of β-catenin (Fig 1A) and higher TCF4 activity than control cell line, Hep3B-pCI (Fig 1B). Hep3B-pCI showed membraneous β-catenin expression without nuclear translocation. Hep3B-S33Y expressed higher GS, Regucalcin, and Lect2 level as compared to Hep3B-pCI, suggesting that these genes are indeed upregulated by *CTNNB1* mutation in human cell line (Fig 1C).

### Relationship between CTNNB1 mutations and β-Catenin target gene expressions

**Fig 2:**
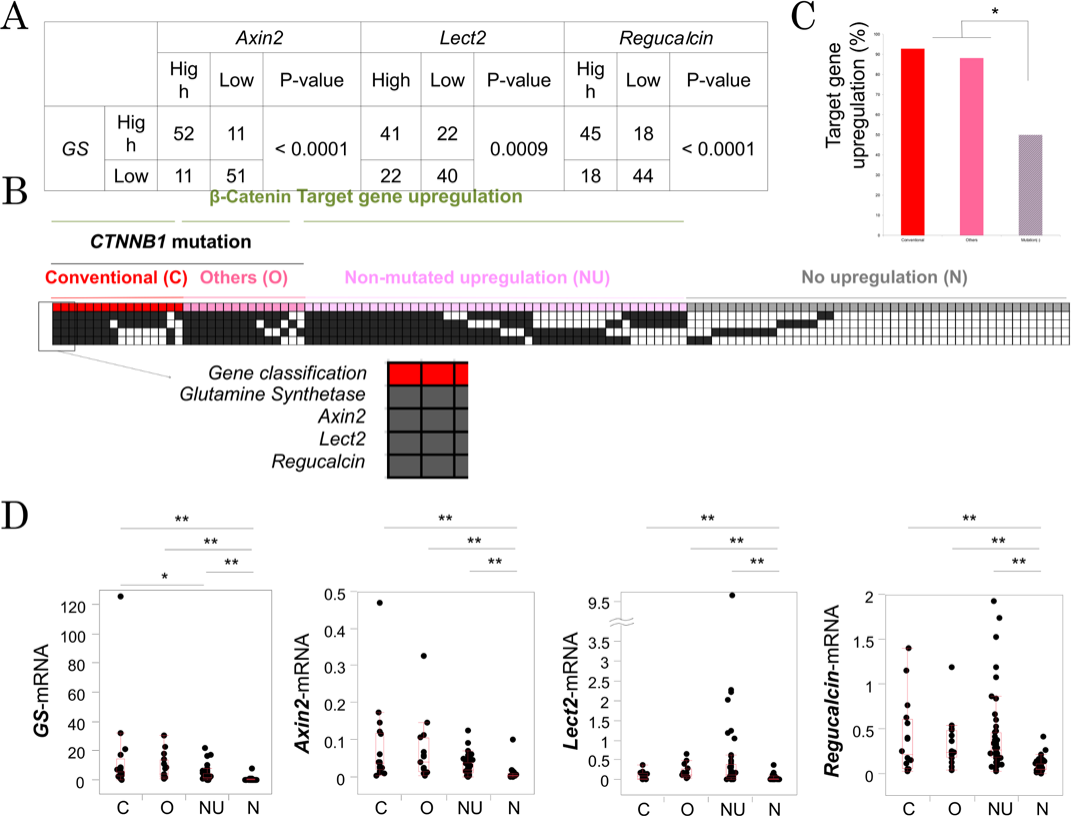
β-catenin signaling based on target gene expressions in human HCC. A. Correlations of β-catenin target gene expressions. B. Heat map is shown based on β-catenin target gene expressions. Patients were classified into 4 groups; patients with conventional mutations (C); patients with other site mutations (O); patients with β-catenin target gene upregulation but no-mutation (NA); patients without mutation or signal activation (N). C. Percentage of β-catenin signal activation is compared among three groups, HCC patients with conventional mutation, patients with other site mutation, and patients without mutations. *p < 0.05. D. quantitative expressions of β-catenin targets, *GS*, *Axin2*, *Lect2*, and *Regucalcin*, are shown. *, *p* < 0.05. **, *p* < 0.01.

To assess the β-catenin activation in human HCC tumors displaying all *CTNNB1* mutations, we examined any co-expression of surrogate Wnt target genes such as *Axin2*, *Regucalcin*, *Lect2* and *GS* using qRT-PCR. Indeed *GS* expression, which is the most reliable target of β-catenin signaling, is significantly correlated with *Axin2* (p<0.0001), *Lect2* (p = 0.0009), and *regucarcin* (p<0.0001) expressions (Fig 2A). Due to known heterogeneity in Wnt signaling in HCC, and with the cut-off value as median value for each gene, we labeled HCC as β-catenin target gene upregulation only if 2 out of 4 Wnt target genes were simultaneously upregulated. Such analysis allowed us to broadly stratify patients into four groups; (1) patients with conventional *CTNNB1* mutations in phosphorylation sites in exon 3 (group C); (2) patients with other non-phosphorylation site *CTNNB1* mutations in exon 3 (group O); (3) patients with wild-type *CTNNB1* but β-catenin target gene upregulation (group NU); and (4) patients with wild-type *CTNNB1* and no activation of β-catenin signaling (group N) (Fig 2B). Intriguingly, almost similar numbers of patients in group C and group O showed active β-catenin signaling (93.8% versus 86.7%), when combined and compared to the rest of HCC patients, rate of β-catenin target gene upregulation in patients who have *CTNNB1* mutations (n = 31) is significantly higher than patients without them (n = 94) (Fig 2C). We also compared expression of each of the four target genes among the four groups. Patients in group N had significantly lower expression of all 4 β-catenin target genes as compared to all other groups (Fig 2D). GS expression in group C was significantly higher than that in group NU and group N. Taken together, gene expression analysis revealed that all exon 3 mutations have remarkable β-catenin activation, and some additional patients without *CTNNB1* mutations also have β-catenin target gene upregulation.

### Clinical relevance of β-catenin activation

**Fig 3:**
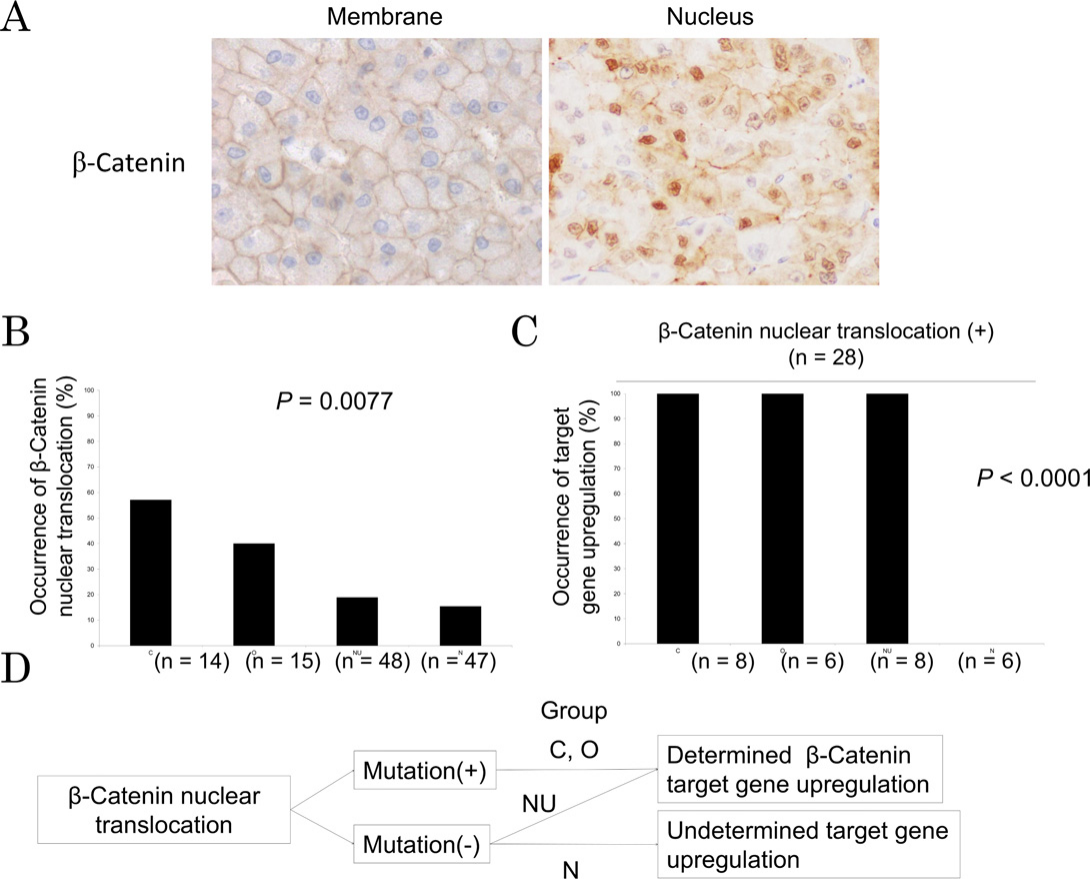
β-catenin signaling assessed by protein expression in human HCC. A. Representative pictures for β-catenin expression in human. B. Occurrence of β-catenin nuclear translocation in each group is shown. C. In patients with β-catenin nuclear translocation (n = 28), occurrence of β-catenin target gene upregulation in each group is shown. D. Patients with β-catenin nuclear translocation in tumor could be divided into two groups; patients with *CTNNB1* mutation and without it. Furthermore, patients without *CTNNB1* mutation were classified into two subgroups; patients with determined β-catenin target gene upregulation and patients without it.

Previous studies regarding immunohistological evaluation of β-catenin activation using expression of GS which is a well-known target of β-catenin suggested that not only HCC with *CTNNB1* mutations but also others without such mutations showed GS overexpression. We aimed to assess β-catenin activation in the two-sided ways by immunohistochemical analysis forcusing on nuclear translocation of β-catenin and by qRT-PCR analysis forcusing on downstream target gene upregulation. Nuclear translocation of β-catenin was seen in 28 patients (22.4%) (Fig 3A and S3 Table). The percentage of nuclear β-catenin translocation was higher in mutated group C and O than non-mutated group NU and N (Fig 3B). Target gene upregulation was 100% seen in patients with β-catenin translocation except for group N where β-catenin translocation was clearly appreciated without downstream described gene upregulation, suggesting that the function of β-catenin is distinct in the group N (Fig 3C). Collecting these clinical observations, some patients in the group N show the distinct role of β-catenin regulating undetermined target genes (Fig 1D).

### Impact of β-catenin activation on prognosis

**Fig 4:**
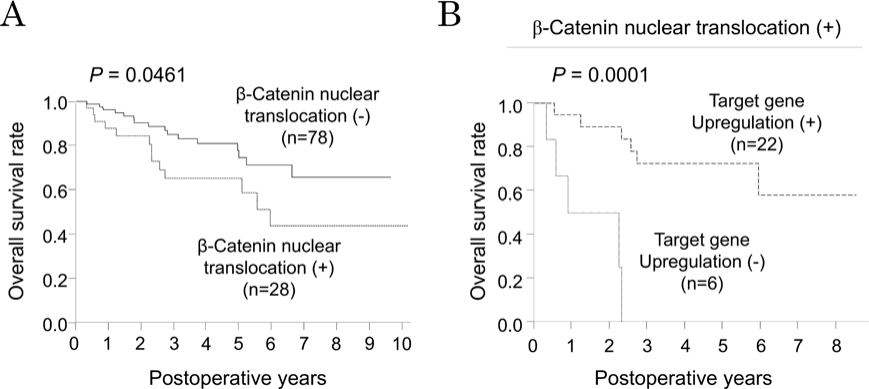
Prognostic impact of β-catenin signaling in human HCC. A. Overall survival is compared between patients with β-catenin nuclear translocation in tumor (n = 28) and patients without it (n = 78). B. Overall survival was compared between patients with described target gene upregulation (n = 22) and others without it (n = 6) in patients with β-catenin nuclear translocation.

Next, we questioned if there were any correlations between aberrant β-catenin signaling and clinical parameters of HCC patients. We compared clinicopathological background of patients in the four groups (S2 Table). There were no significant differences between groups except for high tumor stage evident in the *CTNNB1* other mutation group (*p* = 0.0164). Since there were many patients in our prospective database who received repeated treatments, we only analyzed patients who underwent curative surgery as an initial treatment for HCC in order to more meaningfully determine the prognostic impact of β-catenin signaling. This group of patients showed five year survival rate of 69.1%. There was no difference in prognosis between patients with HCC harboring wild-type or mutated β-catenin gene (*p* = 0.2855). Given the previous reports suggesting that *GS* expression is a good prognostic indicator [14], we first stratified patients based on β-catenin activation as reflected by nuclear β-catenin translocation. Of note, patients with nuclear β-catenin translocation showed significantly worse overall survival than patients without it (*p* = 0.0461) in the current dataset (Fig 4A). Since we found that some patients with nuclear β-catenin translocation are independent of known β-catenin target gene expression, survival analysis was performed only in patients with nuclear β-catenin translocation. Intriguingly, patients without determined target gene upregulation showed remarkably worse survival than others (*p* = 0.0001), suggesting that β-catenin of which function is still undetermined in the current study might have a distinct oncogenic role in this subgroup.

## Discussion

The current study was focused on β-catenin signaling in HCC in clinical settings. We demonstrated that *CTNNB1* exon3 missense mutations not directly affecting phosphorylation sites but adjacent non-phosphorylation codons, have an equally robust effect on β-catenin activation in HCC patients. Gene expression analyses revealed that β-catenin target gene expressions were remarkably similar in these two groups of HCC. Additionally, we reaffirm the existence of a subset where β-catenin signaling is active in absence of *CTNNB1* mutations.

We aimed to determine the significance of mutations in exon 3 of β-catenin gene that did not directly affect a phosphorylation site. Indeed *in vitro* studies have previously addressed the function of some of these mutations [8]. However, no clinical study has directly tested the relevance of these mutations. We show that such mutations indeed activate β-catenin perhaps due to effect on key recognition motifs disallowing binding of kinases such as GSK3β and CK1ε. We found around 50% of all β-catenin mutations to be non-phosphorylation site specific as compared to some previous studies that identified only 33% of patients with β-catenin gene mutations to be of this kind [7].

According to previous observations, the phenotype of β-catenin mutated tumor is mutually exclusive to p53 mutation and characterized by low rate of loss of heterozygosity, less fibrosis in tumor, lacking steatosis, well-differentiated tumor type, and cholestasis [3, 15–17]. Moreover, patients with β-catenin mutated tumors show better prognosis [7, 14]. Our current study did not show any differences in prognosis when patients with mutations in exon 3 of *CTNNB1* were compared to the rest. Instead, when patients were divided into group with or without nuclear β-catenin translocation, the patients with nuclear β-catenin translocation showed a worse overall survival. Among these patients, we unexpectedly found that some patients completely lost described β-catenin target gene upregulation and showed the poorest overall survival. Hoshida et al. recommended that Wnt-TGF-β subclass could be discriminated from *CTNNB1* subclass which contained *CTNNB1*-mutation-signature. In Wnt-TGF-β subclass, most patients showed membraneous β-catenin expression with GS overexpression, suggesting that molecular background of patients with the poorest prognosis in the current study might be distinct from Wnt-TGF-β subclass [18], which is our next issue to be concerned.

Aberrant β-catenin signaling in solid cancers including HCC is well known, and has been discussed as an attractive and therapeutic target to date [9, 10, 19–21]. Preclinical studies suggest that small molecule antagonists of Tcf4/β-catenin complex inhibited the growth of HepG2 cells and downregulated β-catenin target molecule expression, c-myc and cyclin D1, *in vivo* [22]. However, due to other roles of Wnt signaling in liver physiology and a paradoxical increase in chemical carcinogen-induced HCC in β-catenin conditional knockout mice, a careful selection of patients will be relevant [23]. β-catenin mutated tumors should be addicted to β-catenin signaling for tumor progression, and our study demonstrates that both conventional and alternate mutations in exon3 can comparably activate downstream β-catenin target genes. Previous transcriptomic analysis and our current study suggest that patients with β-Catenin signal activation not only in those who have *CTNNB1* mutation but also those who don’t have mutation are presumably candidates for β-Catenin targeted therapy [17, 18]. In addition to the Wnt-TGF-β subclass suggested by Hoshida et al., an another genetic event activating β-Catenin signaling independent of *CTNNB1* mutation is *AXIN1* mutation which is reported to be seen in more than 10% of HCC patients [2]. It is conceivable that they are included in group NU. On the other hand, patients with β-catenin nuclear translocation in the absence of known β-Catenin target gene upregulation showed worse prognosis. The β-Catenin target of those patients remains unexplained. In the previous reports, additional signaling pathways also can converge at β-Catenin. β-Catenin also interacts with transcription factors such as T-cell factor, forkhead box protein O, and hypoxia inducible factor 1α to regulate the expression of target genes [24, 25]. The targets of β-catenin and the mechanism driving tumor progression in those patients carrying with β-catenin nuclear translocation in the absence of *CTNNB1* mutation are issues of great importance to be addressed.

In conclusion, patients with *CTNNB1* exon3 mutation would be desirable candidates for β-Catenin targeted therapy irrespective of mutation cites, and the mechanism of β-Catenin activation in the remaining patients is an urgent issue to be argued. This study would be of great help to understand the β-Catenin activation in patients with HCC.

## Supporting Information

S1 Fig All *CTNNB1* mutations in exon3. (TIF)

S1 Table Somatic mutations of β-Catenin in HCCs. (DOCX)

S2 Table β-Catenin mutation and clinicopathological characteristics. (DOCX)

S3 Table Patient characteristics with β-Catenin nuclear translocation in tumor. (DOCX)

## Abbreviations

HCC: hepatocellular carcinoma
AFP: alpha fetoprotein
PIVKA-II: protein induced by vitamin K absence or antagonist
qRT-PCR: quantitative reverse transcription-polymerase chain reaction
