# Supplementary figures and images for: Diverse Basis of β-Catenin Activation in Human Hepatocellular Carcinoma: Implications in Biology and Prognosis
Source: PLoS One. 2016 Apr 21;11(4):e0152695. doi: 10.1371/journal.pone.0152695 (PMC4839611; doi:10.1371/journal.pone.0152695)

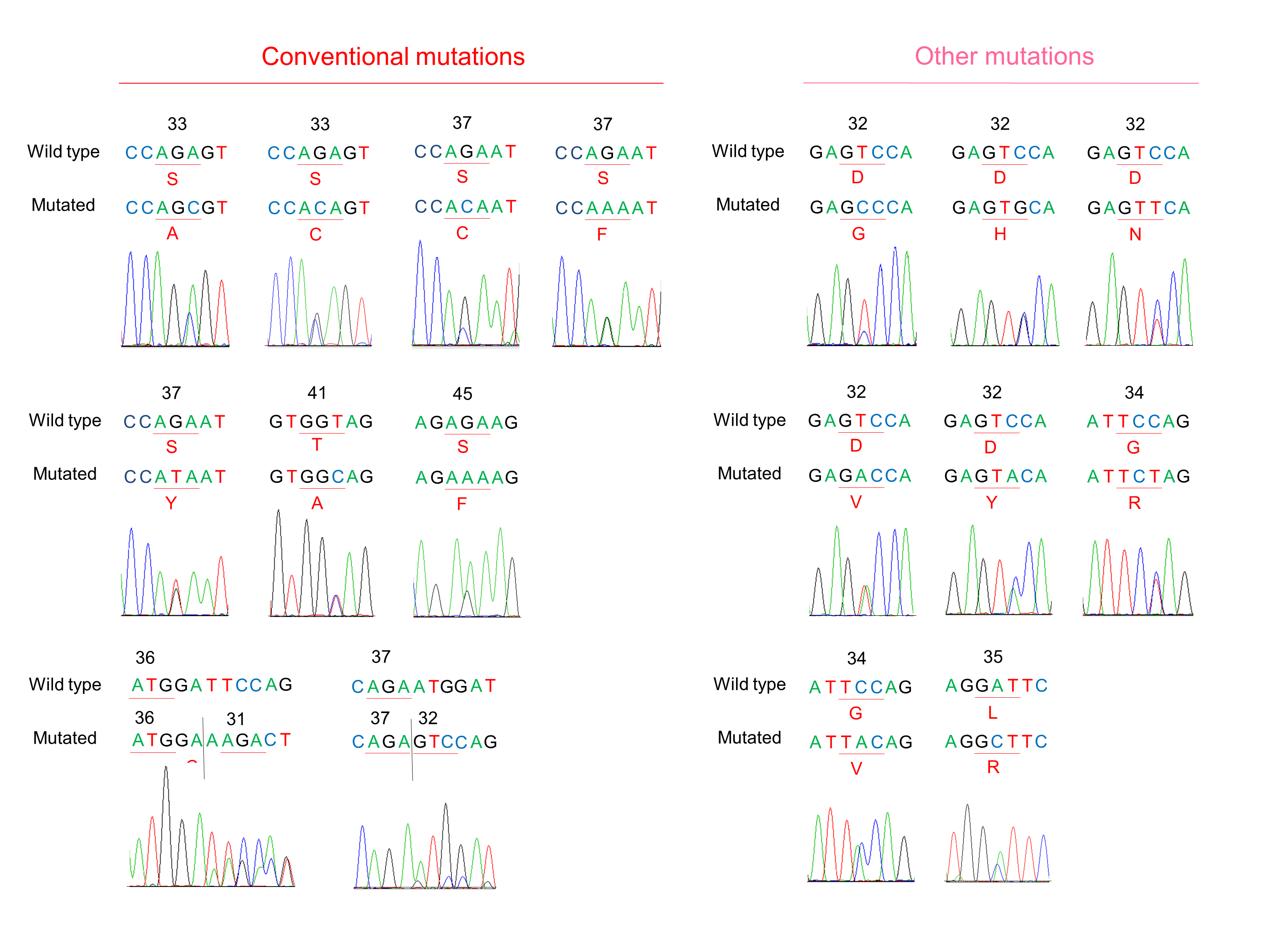

Supplement: S1 Fig — (TIF) [file pone.0152695.s001.TIF]
