# Supplementary material for: Diverse Basis of β-Catenin Activation in Human Hepatocellular Carcinoma: Implications in Biology and Prognosis
Source: PLoS One. 2016 Apr 21;11(4):e0152695. doi: 10.1371/journal.pone.0152695 (PMC4839611; doi:10.1371/journal.pone.0152695)
Supplement: S1 Table — (DOCX) [file pone.0152695.s002.docx]

S1 Table. Somatic mutations of β-Catenin in HCCs

Patients Codon Amino acid Nucleotide

*Conventional (n=16, 12.8 %)*

K310 33 S > A t > g

K26, K35, K41, K127, K148, K302 33 S > C c > g

K27 37 S > C c > g

K318 37 S > F c > t

K140, K320 37 S > Y c > a

K142 41 T > A a > g

K156, K166 45 S > F c > t

K107 del 14 bp including 33-34 codon

K305 del 12 bp including 33-36 codon

*Others (n=15, 12.0 %)*

K138, K160, K165, K304 32 D > G a > g

K150 32 D > H g > c

K59, K93 32 D > N g > a

K101, K159 32 D > V a > t

K71, K121 32 D > Y g > t

K74 34 G > R g > a

K108, K141 34 G > V g > t

K8 35 L > R t > g
