# Supplementary material for: Diverse Basis of β-Catenin Activation in Human Hepatocellular Carcinoma: Implications in Biology and Prognosis
Source: PLoS One. 2016 Apr 21;11(4):e0152695. doi: 10.1371/journal.pone.0152695 (PMC4839611; doi:10.1371/journal.pone.0152695)
Supplement: S2 Table — (DOCX) [file pone.0152695.s003.docx]

S2 Table. β-Catenin mutation and clinicopathological characteristics (n=125)

Clinicopathological factors n β-Catenin status *P*-value

Group C Group O Group NA Group N

n=16 n=15 n=47 n=47

Age* (years) ≥ 66 (%) 71 11 (68.8) 9 (60.0) 24 (51.1) 27 (57.5) 0.6522

Gender Male (%) 105 14 (87.5) 12 (80.0) 38 (80.9) 41 (87.2) 0.5228

AFP† (U/mL) > 20 (%) 47 3 (20.0) 7 (50.0) 22 (47.8) 17 (41.5) 0.2631

PIVKAII* (U/mL)　≥ 136 (%) 49 9 (60.0) 9 (64.3) 15 (32.6) 22 (53.7) 0.0589

Tumor diameter (mm) * ≥ 40 (%) 62 10 (62.5) 6 (40.0) 19 (40.4) 27 (57.5) 0.2253

Tumor number Multiple (%) 36 4 (25.0) 8 (53.3) 11 (23.4) 13 (27.7) 0.1166

Differentiation Poor (%) 20 2 (14.3) 3 (17.7) 4 (8.9) 11 (24.4) 0.2600

Tumor stage III/IVa (%) 70 4 (26.7) 11 (73.3) 15 (31.9) 22 (48.9) 0.0164

Vascular invasion (+)† (%) 50 5 (35.7) 7 (41.2) 16 (34.8) 22 (46.8) 0.6737

HBs-Ag (+) (%) 34 4 (28.6) 5 (29.4) 11 (23.4) 14 (29.8) 0.9064

HCV-Ab (+) (%) 53 7 (43.8) 7 (46.7) 22 (46.8) 17 (36.2) 0.7423

Liver cirrhosis‡ (%) 37 5 (31.3) 5 (33.3) 12 (26.1) 15 (31.9) 0.9170

ΑFP, α-fetoprotein; PIVKA-II, the protein induced by vitamin K absence or antagonist-II; Well, well-differentiated hepatocellular carcinoma; Mod, moderately differentiated hepatocellular carcinoma; HBs-Ag, hepatitis B surface antigen; HCV-Ab, hepatitis C virus antibody

* Cut-off value is defined as the median value.

† Cut-off value is determined by normal value.

‡ Defined by F4 stage from new Inuyama classification (Ichida et al. *Int Hepatol Commun* 1996)
