# Supplementary material for: Diverse Basis of β-Catenin Activation in Human Hepatocellular Carcinoma: Implications in Biology and Prognosis
Source: PLoS One. 2016 Apr 21;11(4):e0152695. doi: 10.1371/journal.pone.0152695 (PMC4839611; doi:10.1371/journal.pone.0152695)
Supplement: S3 Table — (DOCX) [file pone.0152695.s004.docx]

S3 Table. Patient characteristics with β-Catenin nuclear translocation in tumor

Clinicopathological factors β-Catenin nuclear translocation *P*-value

Positive Negative

n=28 n=78

Age* (years) (Median) 66 67 0.8114

Gender Male (%) 21 (77.8) 67 (85.9) 0.3673

AFP† (U/mL) > 20 (%) 10 (40.0) 37 (51.4) 0.3610

PIVKAII* (U/mL)　≥ 136 (%) 13 (48.2) 36 (50.0) 1.0000

Tumor diameter (mm) * ≥ 40 (%) 13 (48.2) 40 (51.3) 0.8259

Tumor number Multiple (%) 13 (48.2) 20 (25.6) 0.0299

Differentiation Poor (%) 1 (11.1) 16 (20.5) 0.2741

Vascular invasion (+)† (%) 12 (44.4) 35 (45.5) 0.9277

HBs-Ag (+) (%) 8 (32.0) 21 (28.4) 0.7308

HCV-Ab (+) (%) 12 (48.0) 29 (39.2) 0.4394

ΑFP, α-fetoprotein; PIVKA-II, the protein induced by vitamin K absence or antagonist-II; Poor, poorly-differentiated hepatocellular carcinoma; HBs-Ag, hepatitis B surface antigen; HCV-Ab, hepatitis C virus antibody

* Cut-off value is defined as the median value.

† Cut-off value is determined by normal value.
